# Graphene-based plasmonic nanocomposites for highly enhanced solar-driven photocatalytic activities

**DOI:** 10.1039/c9ra06273d

**Published:** 2019-09-26

**Authors:** Rania E. Adam, Ebrahim Chalangar, Mahsa Pirhashemi, Galia Pozina, Xianjie Liu, Justinas Palisaitis, Håkan Pettersson, Magnus Willander, Omer Nur

**Affiliations:** Department of Sciences and Technology, Linköping University Campus Norrköping SE-601 74 Norrköping Sweden rania.elhadi.adam@liu.se; School of Information Technology, Halmstad University SE-301 18 Halmstad Sweden; Department of Chemistry, Faculty of Sciences, University of Mohaghegh Ardabili P.O. Box 179 Ardabil Iran; Department of Physics, Chemistry, and Biology (IFM), Linköping University 58183 Linköping Sweden; Solid State Physics and NanoLund, Lund University SE-221 00 Lund Sweden

## Abstract

High-efficiency photocatalysts are crucial for the removal of organic pollutants and environmental sustainability. In the present work, we report on a new low-temperature hydrothermal chemical method, assisted by ultrasonication, to synthesize disruptive plasmonic ZnO/graphene/Ag/AgI nanocomposites for solar-driven photocatalysis. The plasmonic nanocomposites were investigated by a wide range of characterization techniques, confirming successful formation of photocatalysts with excellent degradation efficiency. Using Congo red as a model dye molecule, our experimental results demonstrated a photocatalytic reactivity exceeding 90% efficiency after one hour simulated solar irradiation. The significantly enhanced degradation efficiency is attributed to improved electronic properties of the nanocomposites by hybridization of the graphene and to the addition of Ag/AgI which generates a strong surface plasmon resonance effect in the metallic silver further improving the photocatalytic activity and stability under solar irradiation. Scavenger experiments suggest that superoxide and hydroxyl radicals are responsible for the photodegradation of Congo red. Our findings are important for the fundamental understanding of the photocatalytic mechanism of ZnO/graphene/Ag/AgI nanocomposites and can lead to further development of novel efficient photocatalyst materials.

## Abstract 2

High-efficiency of plasmonic ZnO/graphene/Ag/AgI nanocomposites for solar-driven photocatalysis activities.

## Graphical abstract

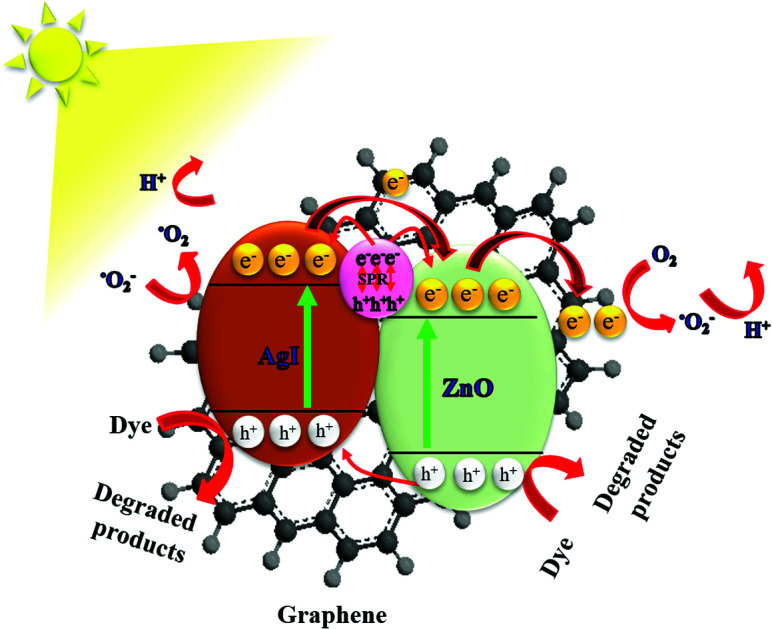

## 1 Introduction

Research on solar energy-driven photocatalysts is growing fast due to environmental concerns and to the need for utilization of sustainable energy resources.^1–4^ So far, semiconductor-based nanostructures are among the most studied materials for photocatalytic purposes due to their remarkably high efficiency.^5–9^ However, many of these semiconductors suffer from issues with poor absorption of light and high recombination rates of photogenerated electron–hole (e^−^/h^+^) pairs.^1,10^ Heterostructure materials offer the possibility of advanced bandgap engineering to drastically improve the scavenging of energy from the sunlight for degradation of harmful organic compounds and contaminants. Various heterostructure materials have been explored with the aim to find materials with enhanced photocatalytic properties under solar light irradiation. Zinc oxide (ZnO) is a remarkable photocatalyst that can be composited with other materials to improve visible light harvesting and, thus, the photocatalytic efficiency. ZnO–graphene nanocomposites are particularly interesting heterostructures with a capability of inhibiting the recombination of photogenerated charge carriers during the photocatalytic process. Graphene (GR) is a well-known 2D material consisting of carbon atoms arranged in a honeycomb lattice structure.^11,12^ It possesses many fundamentally interesting electronic properties such as zero bandgap, zero effective mass, high charge carrier mobility, large surface area, and high optical transparency over a very large spectral range from IR to UV, which makes it an excellent candidate for enhancing the performance of photocatalysts.^10,11,13–15^ It was found that GR and GR oxide can significantly improve the separation efficiency of photogenerated e^−^/h^+^ pairs in photocatalytic processes.^10,11,16–20^ For instance, Tian *et al.*^21^ fabricated ZnO nanorods/reduced GR oxide nanocomposites for enhancing the photodegradation of methylene blue dye. Also, Sawant *et al.*^22^ showed an enhancement of the photodegradation capability of methylene orange and rhodamine B using ZnO/GR nanosheets.

Many different metal–semiconductor nanostructures have been studied as plasmonic photocatalysts.^6,23,24^ Recently, Ag-based semiconductors exhibiting a strong surface plasmon resonance effect (SPR)^23,25–29^ were extensively investigated as visible light photocatalysts for degradation of organic pollutants and toxic dyes. In order to further increase the photocatalytic activity of the ZnO/GR photocatalyst under visible light irradiation, a narrow bandgap silver halide *e.g.* silver iodide (AgI) can be added to the nanocomposite.^25^ The blending of AgI with ZnO shifts the absorption of the nanocomposite significantly towards the visible light region.^11,27,30^ In addition, the excellent conductivity of AgI nanostructures can promote electron transfer that suppresses e^−^/h^+^ recombination and, thus, enhances the interfacial charge transfer.^25^ Therefore, it is expected that the addition of Ag/AgI to ZnO/GR would result in a new nanocomposite with excellent photocatalytic performance. So far, to the best of our knowledge, there are no reports on photocatalytic properties of nanocomposites comprising Ag/AgI and ZnO/GR with respect to degradation of CR dye under solar light irradiation.

In the present work, we report on new plasmonic ZnO/GR/Ag/AgI nanocomposites with highly enhanced photocatalytic capability under simulated solar light irradiation. The nanocomposites were prepared *via* an ultrasonic-assisted hydrothermal solution-based procedure and used for photodegradation of CR dyes. First, ZnO/GR nanocomposites were prepared through hydrothermal growth of ZnO nanoparticles (NPs) on GR nanosheets.^20^ Subsequently, Ag/AgI NPs were grown on the ZnO/GR nanocomposites *via* an ultrasonic irradiation method.^20^ Also, pristine ZnO NPs and ZnO/Ag/AgI nanocomposites were prepared as reference samples. The ZnO/GR/Ag/AgI nanocomposite exhibits superior photocatalytic performance compared to ZnO NPs, ZnO/GR, and ZnO/Ag/AgI nanocomposites. The enhanced photodegradation of CR dyes is primarily attributed to increased light absorption and separation of photogenerated charge carriers by the unique heterojunctions formed between the ZnO, GR, and Ag/AgI counterparts. We present a detailed model of the enhanced photocatalytic mechanism based on the calculated band structure profile of the ZnO/GR/Ag/AgI heterojunctions.

## 2 Experimental part

### 2.1 Materials

All the chemicals used in this work were purchased from Sigma Aldrich and used without any further purification, including the multilayer GR powder, zinc acetate dihydrate (Zn(CH_3_COO)_2_·2H_2_O), potassium hydroxide (KOH), silver nitrate (AgNO_3_), and sodium iodide (NaI). Deionized (DI) water was used in all steps.

### 2.2 Samples preparation

#### 2.2.1 ZnO/GR

The ZnO/GR nanocomposite with a GR-to-ZnO weight ratio of 1 : 99 was synthesized by adding a 10 mg L^−1^ dispersion of GR powder in a zinc acetate dehydrate solution (0.01 M) in DI water. Subsequently, KOH (0.05 M) was added dropwise to the above solution at 60 °C in an ultrasonic bath and kept for 10 minutes. The obtained ZnO/GR nanocomposite was then washed in water and acetone and centrifuged at 3000 rpm for 10 minutes three times, followed by drying in an oven at 120 °C overnight.

#### 2.2.2 ZnO/GR/Ag/AgI

**Fig. 1:**
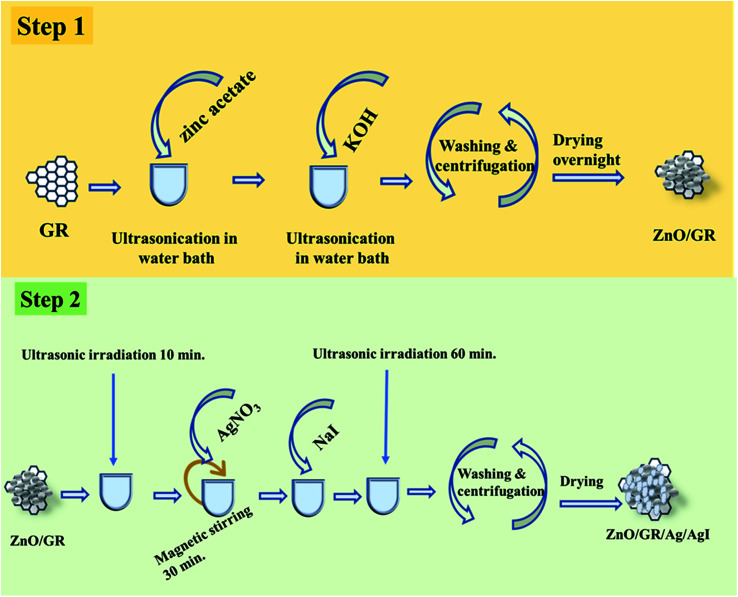
Schematic diagram for preparation of ZnO/GR/Ag/AgI nanocomposites.

To prepare the ZnO/GR/Ag/AgI nanocomposites, Ag/AgI was added to the as-prepared ZnO/GR with three different weight ratios *X* = 10%, 20%, and 30% forming the corresponding nanocomposite denoted ZnO/GR/Ag/AgI (*X*%). First, ZnO/GR was dispersed into 200 mL of DI water with ultrasonic irradiation for 10 minutes and then AgNO_3_ was added to the suspension and stirred for 30 minutes. Next, an aqueous solution of NaI was added dropwise and the final suspension was ultrasonicated for one hour. The nanocomposites were separated by centrifugation and washed two times with DI water and acetone to remove any residuals, and finally dried in an oven at 75 °C for 6 hours. Fig. 1 shows the preparation steps for fabricating the ZnO/GR/Ag/AgI nanocomposites.

### 2.3 Sample characterization

Powder X-ray diffraction (XRD) was used to study the structural properties of the prepared samples using a Philips powder diffractometer (1729 PW) equipped with a Cu K(α) radiation source at 40 kV/40 mA. The sample morphology was investigated with a field-emission scanning electron microscope (FE-SEM), equipped with a Sigma 500 Gemini field emission gun operating at 10 kV. Samples for transmission electron microscopy (TEM) analysis were prepared by dispersing the obtained nanocomposites onto a lacey carbon film suspended by copper TEM grids. Scanning TEM (STEM) high-angle annular dark-field imaging (HAADF), energy-dispersive X-ray spectroscopy (EDS) as well as TEM imaging were performed in the double-corrected Linköping FEI Titan^3^ 60–300, operated at 300 kV. The microscope is equipped with image and probe Cs correctors and a monochromated high brightness XFEG gun and an efficient high solid angle Super-X EDX detector. The chemical composition of the samples was investigated by X-ray photoelectron spectroscopy (XPS) using a Scienta ESCA-200 spectrometer equipped with a monochromatic Al K(α) X-ray source with a photon energy of 1486.6 eV. The optical properties were analyzed by UV-Visible spectrophotometry (PerkinElmer Lambda 900 system) and cathodoluminescence (CL) spectroscopy (Gatan Mono CL4 system combined with FE-SEM). We have also carried out Mott–Schottky tests using a set-up comprising a PGSTAT302N workstation and a standard three-electrode system.

### 2.4 Photocatalytic experiments

The photocatalytic experiments were performed using a solar simulator (100 W ozone-free xenon lamp with a power of 1 sun). In brief, each sample (0.05 g) was mixed with 100 mL of CR dye solution with an initial concentration of 0.02 g L^−1^ and pH ∼ 7 to investigate the photocatalytic activities prior to exposure. These mixtures were then stirred for 30 minutes in dark to reach the adsorption–desorption equilibrium between the photocatalyst and the dye molecules. After a given interval of irradiation time, a small amount of the mixtures was withdrawn and centrifuged. Finally, the UV-Vis absorbance spectra of the CR dye solution were recorded by the UV-Visible spectrophotometer.

## 3 Results and discussion

### 3.1 Analysis of sample characteristics

**Fig. 2:**
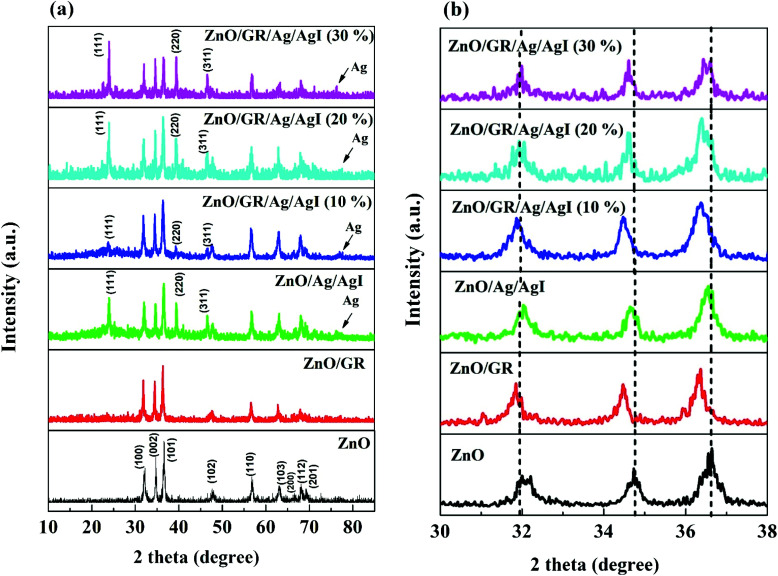
(a) XRD spectra of pristine ZnO NPs, ZnO/GR, ZnO/Ag/AgI, and ZnO/GR/Ag/AgI nanocomposites with a different weight percentage of Ag/AgI. (b) High-resolution XRD patterns revealing composition-dependent diffraction peak shifts.

Fig. 2 shows the XRD spectra of pristine ZnO NPs, ZnO/GR, ZnO/Ag/AgI, and ZnO/GR/Ag/AgI nanocomposites. It can be observed in Fig. 2(a) that the pristine ZnO NPs exhibit characteristic peaks at 2*θ* values of 31.8°, 34.6°, 36.5°, 47.8°, 56.8°, 63.0°, 66.8°, 67.9°, and 69°, which correspond to the crystal planes (100), (002), (101), (102), (110), (103), (200), (112), and (201), respectively, which all belong to the pure hexagonal wurtzite phase of ZnO (JCPDS no. 36-1451). These results strongly imply that the samples are of good crystalline quality and free from defects. Similar XRD diffraction peaks were in fact observed from the ZnO/GR nanocomposites. The fact that no diffraction peaks from carbon species were observed can be attributed to the low amount of GR (1 wt%) in the samples^18,19^ and to the relatively low diffraction intensity of GR.^31^ The presence of GR in the nanocomposites was identified by TEM and XPS analysis (see below). The addition of AgI nanoparticles to the ZnO and ZnO/GR nanocomposites show diffraction patterns at 2*θ* values of 23.9°, 39.2°, and 46.0°, which could be related to the crystal planes (111), (220), and (311), respectively, of the cubic AgI structure (JCPDS no 01-0503).^27^ This conclusion is supported by the observed increase in the intensity of the AgI-related peaks with increasing AgI content. In addition, a small peak at ∼77° was observed in ZnO/Ag/AgI and various ZnO/GR/Ag/AgI nanocomposites, which can be assigned to the reflections of Ag^0^ (JCPDS no 65-2871)^27,32^ produced during the sample preparation. The effect of GR and Ag/AgI on the crystal structure of the pristine ZnO NPs was examined by more accurate XRD measurements in the region of 30–40° as shown in Fig. 2(b). A small shift of the XRD peaks towards smaller angles was not only observed for the ZnO/GR nanocomposites compared to the pristine ZnO NPs, but in fact detected for all GR incorporated nanocomposites. This shift indicates that the lattice constants of ZnO and Ag/AgI NPs have changed due to the presence of the carbonaceous material, in agreement with previous reports.^33,34^ The crystallite size of the NPs can be calculated by the Scherrer equation:^35^
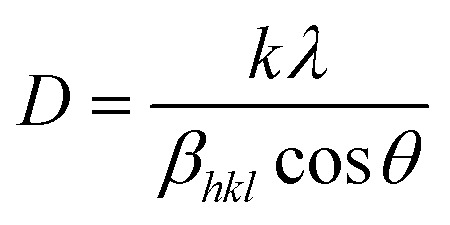
Here *D* is the crystallite size of the ZnO NPs, *λ* is the X-ray wavelength, *θ* is the Bragg diffraction angle, *β* is the full width at half maximum of the diffraction peak corresponding to the (101) plane, and *k* is the Scherrer constant equal to 0.9. It was found that the crystallite size of ZnO NPs is increased slightly after the addition of the Ag/AgI from 17 to 20 nm.

**Fig. 3:**
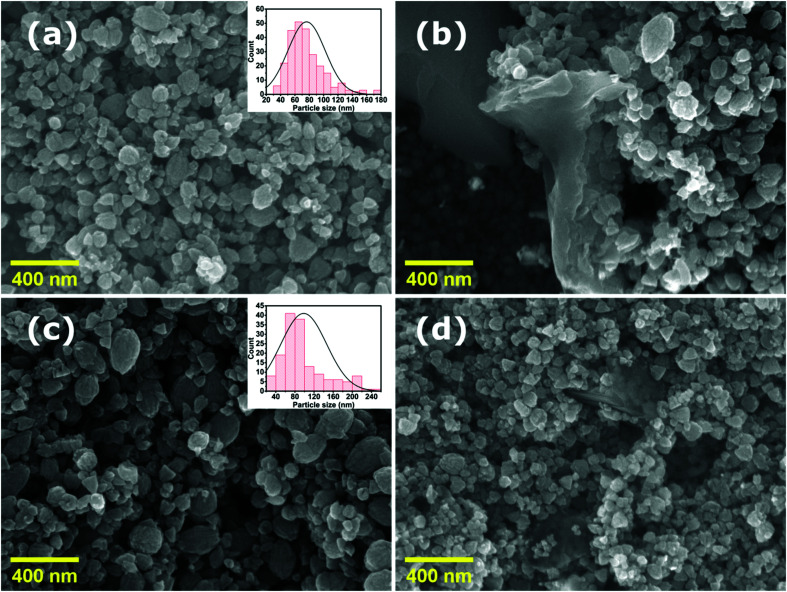
FE-SEM images of (a) pristine ZnO NPs, (b) ZnO/GR, (c) ZnO/Ag/AgI, and (d) ZnO/GR/Ag/AgI (20%) nanocomposites. The insets show the size distribution of the NPs.

Fig. 3 shows FE-SEM images of the synthesized (a) pure ZnO, (b) ZnO/GR, (c) ZnO/Ag/AgI, and (d) ZnO/GR/Ag/AgI (20%) samples. The pure ZnO NPs sample shows a uniform size distribution as compared to the ZnO/Ag/AgI and ZnO/GR/Ag/AgI (20%) nanocomposites. The average size of the ZnO NPs was found to be ∼80 nm, as estimated from the size distribution inset in (a). The average NPs size increased significantly to ∼100 nm (inset in (c)) when Ag/AgI was added to the ZnO NPs, which reflects the formation of ZnO/Ag/AgI agglomerates. The particle size observed in the FE-SEM images is much larger than that extracted from the XRD measurements, indicating a polycrystalline structure of the NPs.

**Fig. 4:**
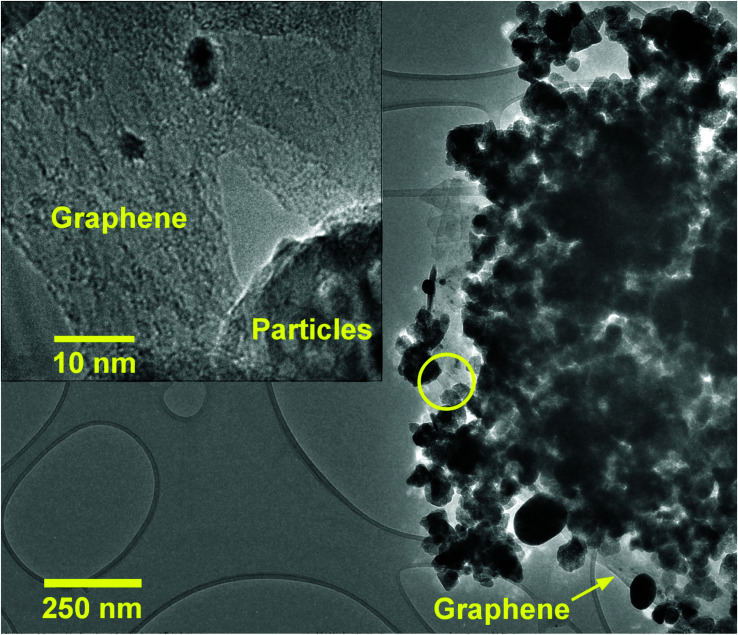
TEM image of ZnO/GR/Ag/AgI (20%) nanocomposites. The inset shows the conjunction of GR and particles (ZnO or Ag/AgI) at higher magnification (region marked by the yellow circle in the main image).

TEM investigations confirm the conjunction of NPs and GR nanoplatelets in ZnO/GR/Ag/AgI (20%) nanocomposites as shown in Fig. 4. The GR nanoplatelets act as a substructure to assemble the nanoparticles enabling an electrical bridge between them. Additionally, it was observed that the GR nanoplatelets have different size and shape caused by the ultrasonic irradiation during the synthesis procedures.

**Fig. 5:**
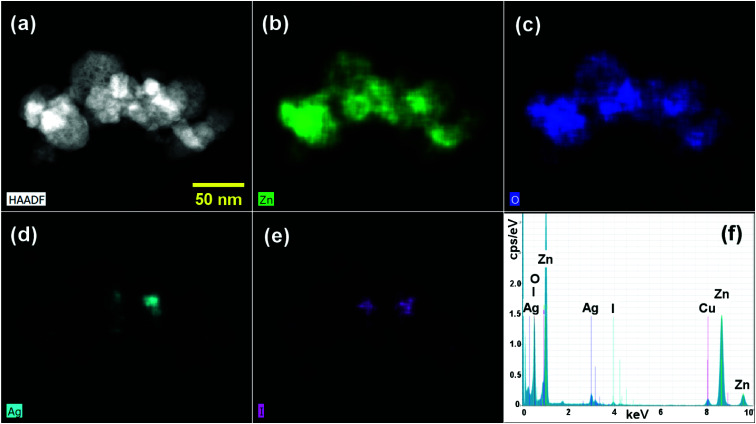
(a) STEM-HAADF image of a ZnO/GR/Ag/AgI (20%) nanocomposite, (b–e) STEM-EDS elemental maps displaying Zn, O, Ag, and I distributions, respectively, and (f) integrated EDS spectra.

The elemental distribution of the ZnO/GR/Ag/AgI (20%) nanocomposites were examined using STEM-HAADF imaging (Fig. 5(a)) and STEM-EDS elemental mapping (Fig. 5(b–e)). The integrated EDS spectra, extracted from EDS maps, confirmed the presence of Zn, O, Ag, and I elements in the nanocomposites. The corresponding elemental EDS maps revealed that the cluster of NPs forming the nanocomposite is mainly composed of ZnO, while Ag/AgI NPs are present in localized areas of the cluster. Carbon mapping did not display any spectral signatures from GR due to overlap with the underlying carbon support grid (not shown). It is worth noting that a better distribution of the elements in the nanocomposites provides a stronger bond between different counterparts. This leads to a more efficient generation and separation of charge carriers under solar irradiation, which in turn leads to excellent photocatalytic activities and higher photodegradation efficiency.

**Fig. 6:**
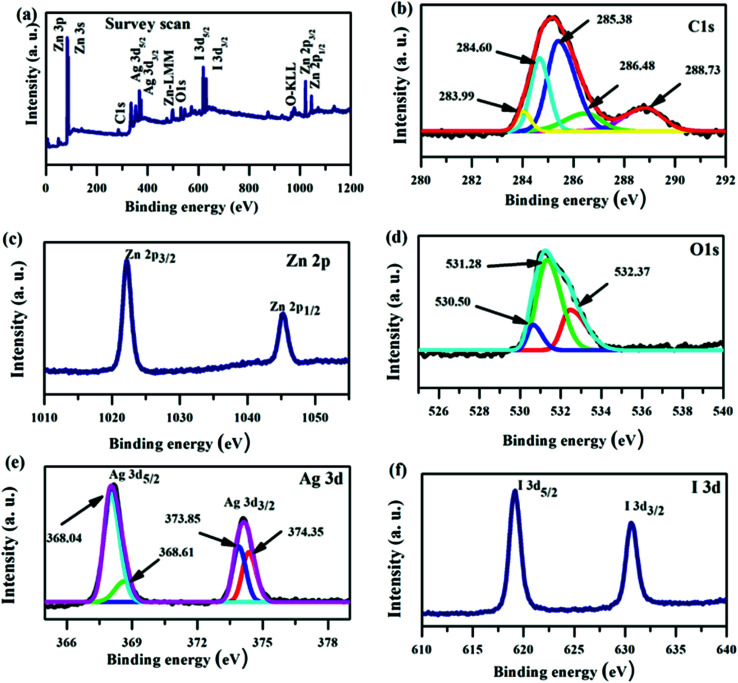
XPS spectra for a ZnO/GR/Ag/AgI (20%) nanocomposite. (a) Survey scan, and high resolution core level spectra of (b) C 1s, (c) Zn 2p, (d) O 1s, (e) Ag 3d, and (f) I 3d.

Furthermore, the chemical oxidation state of the elements in the ZnO/GR/Ag/AgI (20%) nanocomposite was identified by XPS analysis. From the XPS survey scan spectrum in Fig. 6(a) we could clearly identify the existence of Zn, O, C, Ag, and I in the nanocomposite. The high resolution of the C 1s spectrum shown in Fig. 6(b) was deconvoluted into three Gaussian peaks. Besides the C–C bond peak at 284.79 eV, which demonstrates the introduced impurities from the surface contamination,^36^ there are two dominant peaks at 285.57 and 288.71 eV assigned to C–O–C and C=O bonds,^11,36,37^ respectively. As shown in Fig. 6(c), the XPS core level spectrum of Zn 2p is composed of two peaks centered at 1022.16 and 1045.23 eV, which are ascribed to the binding energy of Zn 2p_3/2_ and Zn 2p_1/2_ respectively.^38^ The core-level spectrum of O 1s is presented in Fig. 6(d) and it is divided into two Gaussian fitted peaks. The peak centered at 530.50 eV corresponds to oxygen in the ZnO NPs lattice in the nanocomposite structure.^37,39,40^ The peak at 532.37 eV can be ascribed to oxygen bonded to carbon atoms (the functional group present on GR) and also to the hydroxyl group adhering to the surface of nanocomposite.^41,42^ The XPS spectrum of Ag 3d is shown in Fig. 6(e). The main peaks at 368.13 and 374.06 eV are assigned to Ag 3d_5/2_ and Ag 3d_3/2_, respectively. The Ag 3d_5/2_ is further divided into two peaks at 367.82 and 368.28 eV and the Ag 3d_3/2_ peak is also divided into two peaks at 373.79 and 374.32 eV. The low energy peaks at 367.82 and 373.79 eV are attributed to Ag^+^, whereas the peaks at 368.28 and 374.32 eV are related to metallic Ag^0^.^27,43^ Accordingly, metallic Ag^0^ is produced at the surface of the ZnO/GR/Ag/AgI (20%) photocatalyst during the sample preparation, an observation consistent with the XRD results shown in Fig. 2(a). The chemical state of the I element, which is present in the form of I^−^ ions in the nanocomposite, is revealed in Fig. 6(f). Two clearly resolved peaks related to I 3d_5/2_ and I 3d_3/2_ are located at 619.02 and 630.58 eV, respectively.^30^

**Fig. 7:**
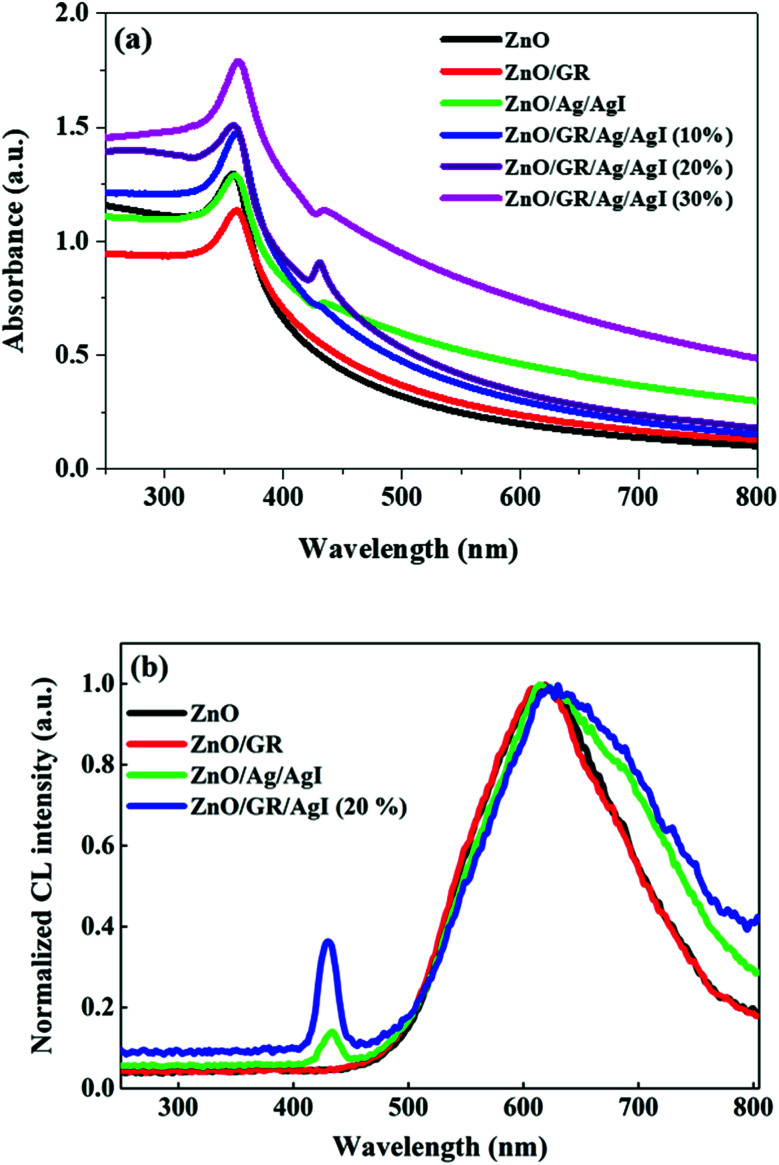
(a) UV-Vis absorbance spectra of pristine ZnO NPs, ZnO/GR, ZnO/Ag/AgI, and ZnO/GR/Ag/AgI nanocomposites with a different weight percentage of Ag/AgI, and (b) corresponding CL spectra.

The optical characteristics of the nanocomposites were examined by UV-Vis spectroscopy and CL measurements. The UV-Vis absorbance spectra of ZnO NPs, ZnO/GR, ZnO/Ag/AgI, and ZnO/GR/Ag/AgI nanocomposites with different weight percent of Ag/AgI are shown in Fig. 7(a). It is clear that pure ZnO NPs exhibit a strong interband absorption edge around 400 nm. The weak absorbance in the visible region is attributed to the presence of native point defects.^29^ The addition of GR to ZnO slightly redshifts the absorbance edge. This redshift can be explained by the influence of GR on the surface electric charge in the ZnO/GR photocatalyst.^10,44^ In the case of ZnO/GR/Ag/AgI nanocomposites, a broader absorbance is observed in the visible region (*λ* > 400 nm) which increases with the weight percent of Ag/AgI. Therefore, the blending of Ag/AgI into the nanocomposite can increase its photocatalytic performance under solar irradiation. The observed absorbance peak at *λ* = 430 nm is assigned to the surface plasmon absorption of the Ag/AgI NPs, indicating the presence of Ag^0^ at the surface of the nanocomposites.^45^ As a result, due to contributions from different counterparts, the fabricated plasmonic nanocomposite displays the highest optical absorbance, which is beneficial for the photocatalytic performance under solar irradiation. Fig. 7(b) shows CL spectra of ZnO, ZnO/GR, ZnO/Ag/AgI, and ZnO/GR/Ag/AgI (20%) samples, measured at room temperature. Interestingly, all samples exhibit a broad emission band covering the whole visible region with a luminescence peak centered at ∼600 nm. This band can be attributed to radiative transitions associated with intrinsic deep levels such as oxygen vacancies and interstitials (V_O_, O_i_), as well as to the presence of hydroxyl groups at the surface of the ZnO NPs.^46–48^ In addition, the observed peak at *λ* = 430 nm is attributed to surface plasmon-enhanced luminescence, induced by the Ag/AgI NPs, in agreement with the UV-Vis spectrum in Fig. 7(a). The ZnO/GR/Ag/AgI (20%) nanocomposite exhibits broader band luminescence than that of the pure ZnO NPs and ZnO/GR, again consistent with the results presented in Fig. 7(a). Evidently, the presence of Ag/AgI has a significant positive effect on the emission properties in the visible region, which in turn is expected to improve the photocatalytic capability of the nanocomposite.

### 3.2 Photocatalytic performance

#### 3.2.1 Photodegradation of toxic wastewater

**Fig. 8:**
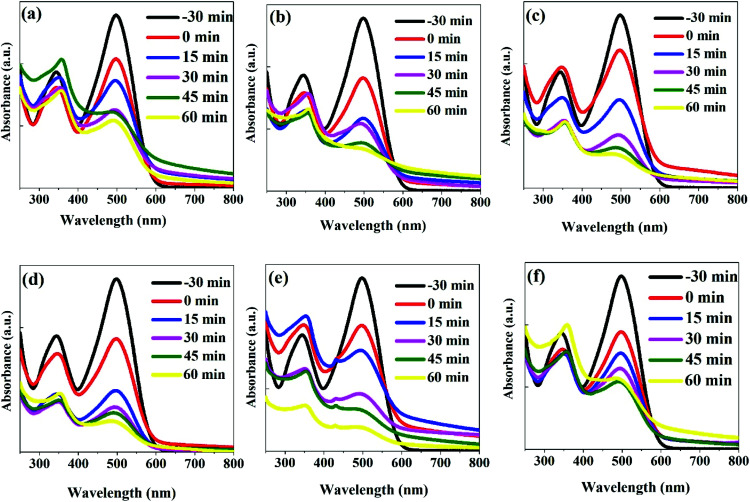
UV-Vis absorbance spectra of CR dyes recorded after different photodegradation time intervals using (a) ZnO, (b) ZnO/GR, (c) ZnO/Ag/AgI, (d) ZnO/GR/Ag/AgI (10%), (e) ZnO/GR/Ag/AgI (20%), and (f) ZnO/GR/Ag/AgI (30%) photocatalysts. The spectra marked by “−30 min” were recorded for the CR only. The spectra marked by “0 min” were recorded for the samples kept in darkness for 30 min (no irradiation).

We studied the photocatalytic activities of ZnO, ZnO/GR, ZnO/Ag/AgI and ZnO/GR/Ag/AgI samples through the degradation of CR dye under simulated solar irradiation. Fig. 8(a–f) shows the recorded UV-Vis absorbance of the CR dye mixed with the nanocomposite in 15 min steps of up to 60 min irradiation. First, the nanocomposite/CR dye was kept in dark for 30 min to reach the adsorption–desorption equilibrium state between the photocatalysts and CR molecules. Then the light was turned on, and the absorbance spectra were recorded after each 15 min step for all samples. It is well-known that carbon-based materials have an excellent adsorption capacity. This plays a key role in the present nanocomposites where the enhanced dye adsorption on the surface of the photocatalysts strongly increases their photocatalytic performance.^49^ With increasing irradiation time, the main absorbance peak at 497 nm related to CR reduces progressively, verifying the degradation of the CR dye. After 60 min irradiation, the absorbance peak for the ZnO/GR/Ag/AgI (20%) nanocomposite has almost disappeared (Fig. 8(e)). In contrast, the variation of the absorbance peaks for the ZnO, ZnO/GR, and ZnO/Ag/AgI samples were small as shown in Fig. 8(a, b, and c), respectively. The enhanced photocatalytic performance of ZnO/GR/Ag/AgI nanocomposites can be assigned to the synergistic effect of ZnO/GR and Ag/AgI, and the improved charge transfer ability of the photo-generated carriers at their interfaces as discussed in detail below.

The photodegradation efficiency (%) was calculated according to eqn (1) and the results are shown in Fig. 9.1
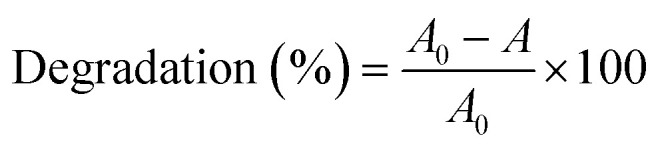
where *A*_0_ is the initial absorbance of the dye, and *A* is the absorbance after a specific irradiation time.

**Fig. 9:**
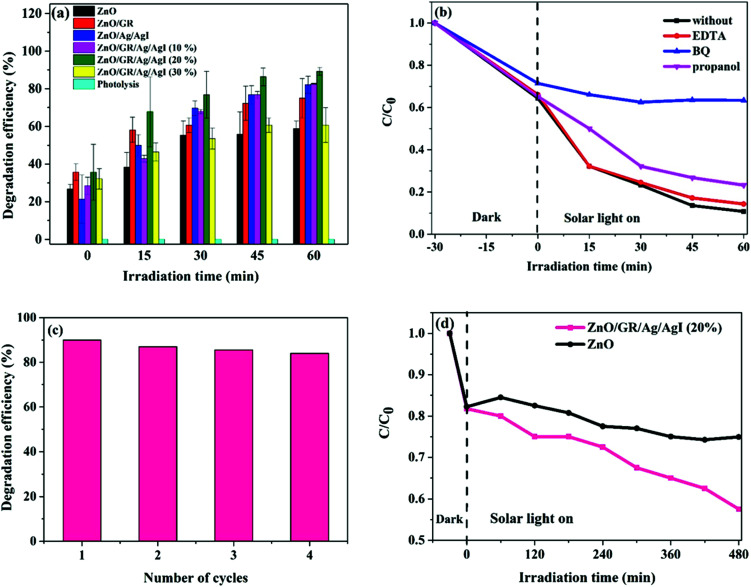
(a) Photodegradation efficiency of CR dyes using ZnO, ZnO/GR, ZnO/Ag/AgI and ZnO/GR/Ag/AgI photocatalysts with different weight percentage of Ag/AgI, (b) photocatalytic activities of CR with ZnO/GR/Ag/AgI (20%) nanocomposite in the presence of different scavengers, (c) recycling of ZnO/GR/Ag/AgI (20%) nanocomposite during degradation of CR, and (d) photodegradation of phenol with ZnO and ZnO/GR/Ag/AgI (20%) nanocomposite.

Fig. 9(a) displays the calculated degradation efficiency of the ZnO, ZnO/GR, ZnO/Ag/AgI, and ZnO/GR/Ag/AgI samples under solar irradiation. In the absence of any photocatalyst, the photolysis of the CR is negligible, which shows that this pollutant has high stability during extended periods of irradiation. In contrast, the usage of ZnO NPs and ZnO/GR nanocomposites enhance the photocatalytic performance. In particular, the incorporation of GR leads to a significantly higher photodegradation efficiency of 75% compared to 58% for pure ZnO NPs, an effect primarily attributed to inhibition of the recombination of photogenerated e^−^/h^+^ pairs during the photocatalytic reaction. However, in comparison to pure ZnO NPs, and ZnO/GR, the plasmonic ZnO/Ag/AgI and ZnO/GR/Ag/AgI nanocomposites show superior photocatalytic activities for CR photodegradation. This important result originates first of all from the addition of Ag/AgI which increases the absorption of photons, and thus, the number of photogenerated e^−^/h^+^ pairs in the visible region of the solar spectrum. Interestingly, the photocatalytic performance of the plasmonic nanocomposites depends strongly on the Ag/AgI weight ratio. The ZnO/GR/Ag/AgI (20%) nanocomposite exhibits the highest photodegradation efficiency reaching 90% after 60 min of irradiation. However, with increasing Ag/AgI weight ratio up to 30%, the photodegradation efficiency decreased to only 60% after 60 minutes of irradiation. This drop in efficiency can be explained by the excess of Ag/AgI acting as efficient recombination centers. Moreover, the Ag/AgI species might cover the active sites on the nanocomposite surface and thereby passivate the effective junctions between the counterparts which lead to a reduced charge separation efficiency in ZnO/GR/Ag/AgI (30%) sample. A specific content of Ag/AgI is thus crucial for optimizing the photocatalytic performance of plasmonic ZnO/GR/Ag/AgI nanocomposites.

The photodegradation of the CR dye may follow two different processes of direct mineralization or stepwise *N*-deethylation. The direct mineralization involves gaseous end-products such as CO_2_, NO_3_, and NO_*x*_, whereas the stepwise *N*-deethylation leads to a destruction of the conjugated structure of the CR dye which results in a complete degradation. In our photodegradation experiments, the absorption of the CR is decreasing with time without any shift in the absorbance spectrum after 60 min of irradiation (Fig. 8(e)). This suggests that the CR dye was directly mineralized, producing end-products of CO_2_, NO_3_, and NO_*x*_.^15,50,51^

To further reveal the photoreactivity mechanisms involved in photodegradation of the CR dye with the ZnO/GR/Ag/AgI (20%) nanocomposite, trapping experiments were performed to identify the reactive species that are involved in the photocatalytic mechanisms. For this purpose, ethylenediaminetetraacetic acid (EDTA), benzoquinone (BQ) and 2-propanol were used as scavengers for h^+^, superoxide radicals, and hydroxyl radicals, respectively.^52–55^ As shown in Fig. 9(b), the photodegradation process was suppressed with the addition of BQ due to the quenching of the superoxide radicals, indicating that the superoxide radicals are the main reactive species involved in the photodegradation of the CR dye. Also, it was observed that the addition of 2-propanol showed partial inhabitation of the degradation of the CR, suggesting that the hydroxyl radicals also play an important role for the photocatalytic performance. Surprisingly, the addition of EDTA did not have any noticeable effect at the beginning of the degradation process. However, the quenching of holes due to the addition of the EDTA gradually increased after 30 min of irradiation.

To further investigate the photocatalytic stability and reusability of the ZnO/GR/Ag/AgI (20%) nanocomposite, recycling tests of the efficiency for photodegradation of CR were performed. After four successive degradation experiments (Fig. 9(c)), only a small reduction in the efficiency was observed, indicating an excellent stability of the nanocomposite.

To rule out any effects of photosensitization of the CR dye under visible light, the photocatalytic performance of the various nanocomposites in this work was investigated for colourless phenol (0.02 g L^−1^), well known to be insensitive to visible light. A mixture of 100 mL of a phenol solution with 0.05 g of the respective nanocomposite was stirred for 30 min in dark to reach the adsorption–desorption equilibrium. Then UV-Vis absorbance spectra of the different mixtures were recorded in 60 min time intervals. The absorption peak of phenol at 270 nm was used to calculate the concentration of phenol at each time (Fig. 9(d)). It is obvious that the concentration of phenol is decreasing gradually with irradiation time, indicating the decomposition of phenol. In comparison to pure ZnO NPs, the ZnO/GR/Ag/AgI (20%) nanocomposite shows a superior efficiency for photodegradation of phenol. This result also implies that the effect of dye sensitization on the degradation efficiency is small for both phenol and CR.

**Fig. 10:**
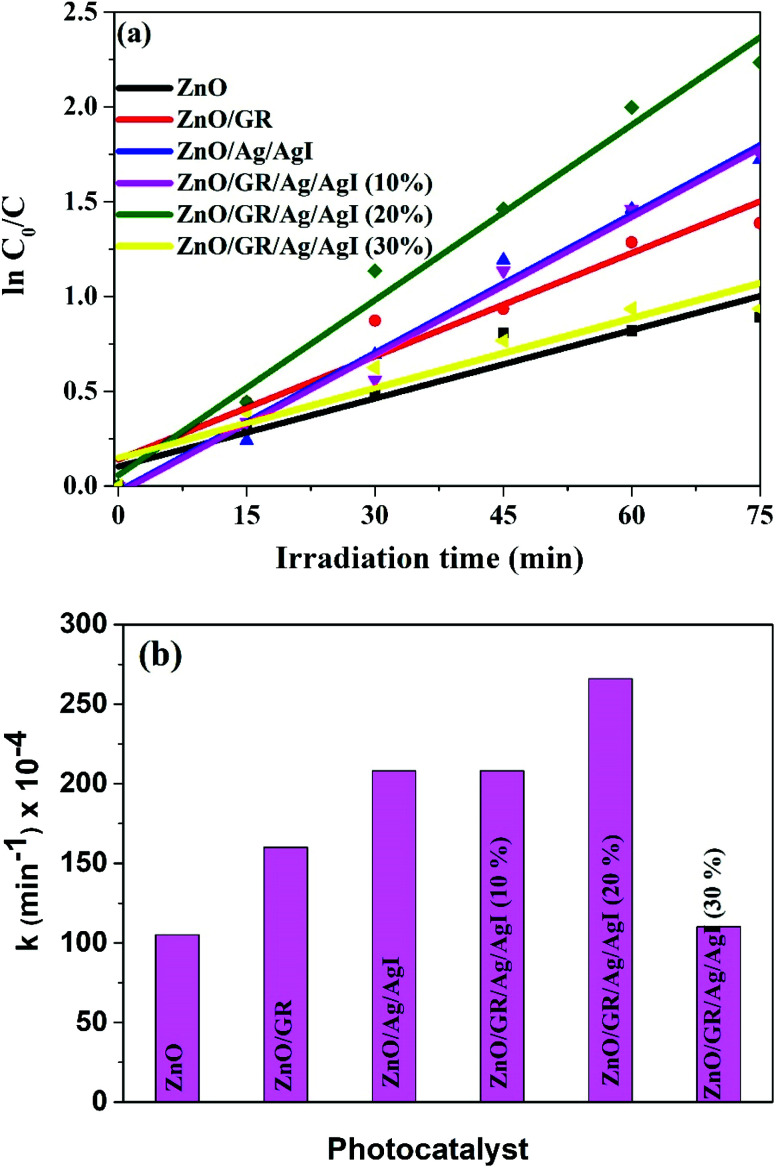
(a) ln(*C*_0_/*C*) *versus* irradiation time, and (b) degradation rate constant of CR dyes using ZnO, ZnO/GR, ZnO/Ag/AgI and ZnO/GR/Ag/AgI photocatalysts with a different weight percentage of Ag/AgI.

To develop a deeper understanding of the photocatalytic mechanisms, the apparent reaction constant for each photocatalyst was investigated according to the Langmuir–Hinshelwood's pseudo-first order kinetics model^56^ shown in eqn (2) below.2
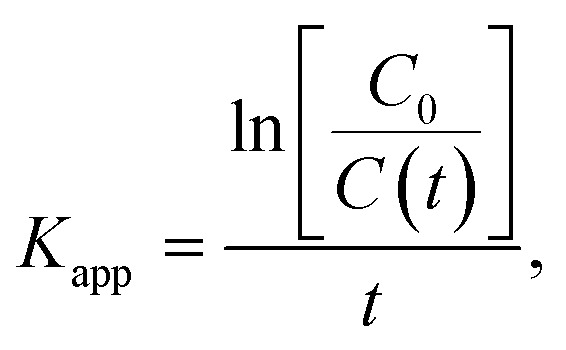
where *K*_app_ is the apparent reaction constant (min^−1^), *t* is the irradiation time (min), *C*_0_ is the initial concentration mol L^−1^ of the dye, and *C*(*t*) is the concentration mol L^−1^ of the dye after irradiation time *t*. The plot of 
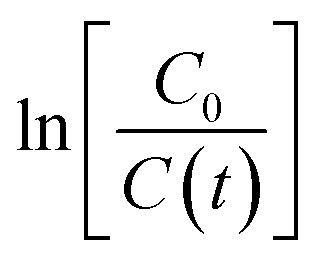
*versus* irradiation time is shown in Fig. 10(a) and found to be in excellent agreement with the Langmuir–Hinshelwood model. The degradation rate constant of CR is found to be 105 × 10^−4^, 160 × 10^−4^, 208 × 10^−4^, 208 × 10^−4^, 266 × 10^−4^, and 110 × 10^−4^ min^−1^ for ZnO, ZnO/GR, ZnO/Ag/AgI, ZnO/GR/Ag/AgI (10%), ZnO/GR/Ag/AgI (20%), and ZnO/GR/Ag/AgI (30%), respectively. It is clear that the degradation rate constant for ZnO/GR/Ag/AgI (20%) is superior to those of the other GR-based nanocomposites.

In Table 1 we show a comparison of the degradation efficiency of different reported GR-based photocatalysts. Summarized, this survey demonstrates that the present plasmonic ZnO/GR/Ag/AgI (20%) nanocomposite has very high photodegradation efficiency with respect to the irradiation time of the photocatalytic reaction and with reasonable degradation rate constant.

**Table 1:** Comparison of state-of-the-art GR-based nanocomposites for photodegradation of organic dyes

Photocatalyst	Synthesis method	Light source	Dye	Degradation min/efficiency	*K*_app_ (10^−2^ min^−1^)	Year
AgI–mesoTiO_2_/4 wt% RGO	Hummers and ultrasound	150 W tungsten lamp	Methylene orange	90/92%	—	2016 (ref. 11)
P25/1 wt% GR	Modified Hummers method	500 W xenon lamp	Methylene blue	65/65%	—	2010 (ref. 60)
ZnSe/1 wt% GR	Modified Hummers method	Solar simulator with a power of 1 sun	Methylene blue	240/99.5%	1.27	2015 (ref. 61)
Ag/Ag_2_CO_3_–1 wt% RGO	Physical mixing and chemical bath deposition	350 W xenon lamp	Methylene orange	15/7%	1.80	2016 (ref. 62)
BiVO_4_/5.5 wt% RGO/Bi_2_O_3_	Chemical bath deposition	150 W metal halide lamp	Bisphenol A	240/60%	0.031	2017 (ref. 63)
Ag/ZnO/1 wt% GR	Low-temperature microwave-assisted solution	300 W high-pressure Hg lamp	Methylene orange	80/99.6%	0.5	2015 (ref. 64)
1.78 wt% RGO–ZnO	Chemical bath deposition	400 W Hg lamp	Rhodamine B	60/61.5%	—	2015 (ref. 65)
3 wt% GR/ZnO	Hydrolysis deposition	3 W LED lamp	X3B	45/80%	0.34	2015 (ref. 66)
ZnO/10 wt% RGO	Hummers and arc discharge	90 W halogen light	Methylene orange	90/100%	0.47	2015 (ref. 67)
1 wt% GR/ZnO	Hydrothermal	250 W mercury lamp	Methylene blue	450/90%	—	2016 (ref. 68)
Ag_2_CrO_4_–1 wt% GR	Precipitation	300 W xenon lamp	Methylene blue	15/50%	2.80	2015 (ref. 69)
ZnO/1 wt% GR/Ag/AgI	Ultrasonic-assisted hydrothermal	Solar simulator (100 W xenon lamp with a power of 1 sun)	Congo red	60/90%	2.66	Current work

#### 3.2.2 Proposed photocatalytic mechanism

**Fig. 11:**
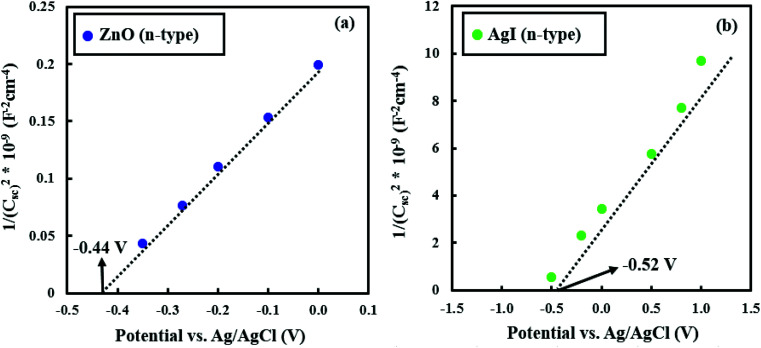
Mott–Schottky plots for (a) ZnO and (b) AgI samples.

To experimentally probe the electronic band structure of ZnO/Gr/Ag/AgI nanocomposites, Mott–Schottky analysis of both ZnO and AgI semiconductors were performed to determine the conduction and valence band edge energies (*E*_CB_ and *E*_VB_).^57,58^ As readily observed in Fig. 11(a and b), both the ZnO and AgI samples exhibit capacitance *vs.* potential characteristics consistent with n-type semiconductors. The flat band potentials can be determined from the extrapolation of 1/*C*^2^ to zero, which found to be −0.44 V and −0.52 V *vs.* Ag/AgCl (*i.e.*, −0.24 V and −0.32 V *vs.* NHE) for ZnO and AgI semiconductors, respectively. It is worth noted that the flat band potential for n-type semiconductor is placed at 0.1 eV more positive potential than the *E*_CB_. Accordingly, *E*_CB_ for the ZnO and AgI was obtained to be −0.34 and −0.42 eV, respectively. In addition, considering the band gap values and based on Mullikan theory calculations,^59^ the *E*_VB_ of ZnO and AgI was computed to be +2.86 eV and +2.37 eV, respectively.

**Fig. 12:**
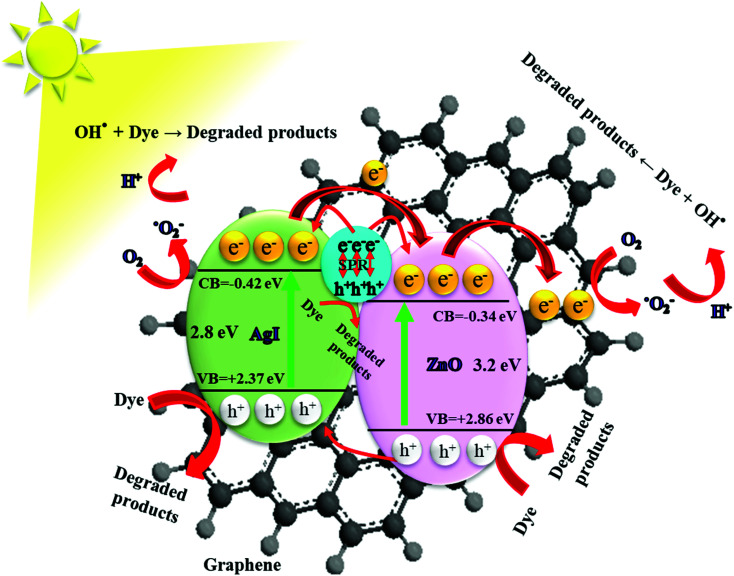
Schematic diagram showing the energy band structure and possible e^−^/h^+^ pair separation and charge carrier transport mechanisms in ZnO/GR/Ag/AgI nanocomposites.

In Fig. 12 we show a schematic of the proposed photocatalytic mechanism deduced from the combined results of the different measurements and analysis methods discussed in this work. In the presence of solar light, ZnO and AgI simultaneously play a key role in absorbing the solar light. Especially AgI, with its smaller bandgap than ZnO, can efficiently increase the absorption in the visible light region.^27^ When solar light impinges on the ZnO/GR/Ag/AgI photocatalyst surface, electrons, and holes are generated in the conduction band (CB) and valence band (VB) in ZnO and AgI, respectively. Owing to the band offsets, the electrons residing in the CB of AgI migrate to the CB of ZnO and, in a likewise manner, holes in the VB of ZnO move to the VB of AgI. This leads to an efficient charge carrier separation and transportation which mitigates the undesired recombination of charge carriers. In addition, the plasmonic Ag NPs act as efficient generators of electrons and holes when excited by visible light *via* the SPR effect. These generated electrons are subsequently injected into the CB of AgI and ZnO, respectively.^15,37^ Subsequently, the photogenerated electrons are transferred from the CB of ZnO to the GR. The GR plays a dual role of electron acceptor and transporter, which improve the photocatalytic behavior of the nanocomposite under solar irradiation. The efficient transfer of electrons and the reduction of the recombination rate at the surface of the photocatalyst strongly enhance the photocatalytic efficiency. The trapped electrons react with oxygen molecules to produce superoxide radicals that subsequently get converted into hydroxyl radicals through multi-electron reduction reactions. Finally, these active species can react with pollutant (dye) molecules and decompose them into H_2_O, CO_2_, and mineral compounds. The photoinduced holes can directly oxidize the adsorbed dye molecules to produce degraded products. As a result, the high photodegradation efficiency of ZnO/GR/Ag/AgI nanocomposites is related to the uniform distribution of the nanoparticles at the surface of the GR nanosheets, and strong synergy effects between GR, ZnO NPs, and Ag/AgI that favours interfacial charge transfer and prevents e^−^/h^+^ recombination.

## 4 Conclusion

In summary, we have reported on a novel low-temperature chemical route, assisted by ultrasonication, to prepare ZnO/GR/Ag/AgI nanocomposites for state-of-the-art photocatalytic performance for degradation of CR and phenol. The prepared plasmonic nanocomposites have been characterized by various techniques to unravel their structural, morphological, electrical, optical and photocatalytic properties. The synthesized ZnO/GR/Ag/AgI (20%) nanocomposite exhibits the highest photodegradation efficiency (90%) and the highest degradation constant rate (308 × 10^−4^ min^−1^) for degradation of CR dye. The key advantages of the optimized nanocomposites include increased light absorption in the visible range due to a strong plasmonic resonance effect involving metallic Ag^0^, and to the improved electronic transfer due to hybridization with graphene. In addition, the nanoparticles in the plasmonic nanocomposite facilitate an effective charge separation of the photoexcited charge carriers, mitigating e^−^/h^+^ pair recombination. The scavenger experiments suggest that superoxide and hydroxyl radicals are responsible for the photodegradation of CR. The recycling test confirms a sufficient durability of the nanocomposite. We believe that the proposed plasmonic nanocomposites are promising for high photocatalytic performance applications such as removal of organic and inorganic pollutants, photoelectrochemistry and water oxidation.

## Conflicts of interest

There are no conflicts of interest to declare.

## Supplementary Material
